# The Impact of Serum Parameters Associated with Kidney Function on the Short-Term Outcomes and Prognosis of Colorectal Cancer Patients Undergoing Radical Surgery

**DOI:** 10.1155/2023/2017171

**Published:** 2023-02-27

**Authors:** Bin Zhang, Xu-Rui Liu, Xiao-Yu Liu, Bing Kang, Chao Yuan, Fei Liu, Zi-Wei Li, Zheng-Qiang Wei, Dong Peng

**Affiliations:** ^1^Department of Gastrointestinal Surgery, The First Affiliated Hospital of Chongqing Medical University, Chongqing 400016, China; ^2^Department of Clinical Nutrition, The First Affiliated Hospital of Chongqing Medical University, Chongqing 400016, China

## Abstract

**Purpose:**

The current study was designed to investigate the impact of blood urea nitrogen (BUN), serum uric acid (UA), and cystatin (CysC) on the short-term outcomes and prognosis of colorectal cancer (CRC) patients undergoing radical surgery.

**Methods:**

CRC patients who underwent radical resection were included from Jan 2011 to Jan 2020 in a single clinical centre. The short-term outcomes, overall survival (OS), and disease-free survival (DFS) were compared in different groups. A Cox regression analysis was conducted to identify independent risk factors for OS and DFS.

**Results:**

A total of 2047 CRC patients who underwent radical resection were included in the current study. Patients in the abnormal BUN group had a longer hospital stay (*p*=0.002) and more overall complications (*p*=0.001) than that of the normal BUN group. The abnormal CysC group had longer hospital stay (*p* < 0.01), more overall complications (*p*=*p* < 0.01), and more major complications (*p*=0.001) than the normal CysC group. Abnormal CysC was associated with worse OS and DFS for CRC patients in tumor stage I (*p* < 0.01). In Cox regression analysis, age (*p* < 0.01, HR = 1.041, 95% CI = 1.029–1.053), tumor stage (*p* < 0.01, HR = 2.134, 95% CI = 1.828–2.491), and overall complications (*p*=0.002, HR = 1.499, 95% CI = 1.166–1.928) were independent risk factors for OS. Similarly, age (*p* < 0.01, HR = 1.026, 95% CI = 1.016–1.037), tumor stage (*p* < 0.01, HR = 2.053, 95% CI = 1.788–2.357), and overall complications (*p*=0.002, HR = 1.440, 95% CI = 1.144–1.814) were independent risk factors for DFS.

**Conclusion:**

In conclusion, abnormal CysC was significantly associated with worse OS and DFS at TNM stage I, and abnormal CysC and BUN were related to more postoperative complications. However, preoperative BUN and UA in the serum might not affect OS and DFS for CRC patients who underwent radical resection.

## 1. Introduction

Colorectal cancer (CRC) is the second most fatal tumor worldwide, and it was estimated that nearly 9.4% of cancer-related deaths would be caused by CRC in 2020 [1–3]. The most effective method for the therapy of CRC is still radical surgery [4–6]. Although great progress was made in the surgical techniques, the prognosis of these patients varied for different reasons, such as tumor stage [7, 8], comorbidities [9–11], and complications [12, 13]. For better clinical decisions and to improve the survival of CRC patients, many biochemical indicators, such as albumin [14, 15] and bilirubin [16, 17], were identified to find patients with high risks of postoperative complications and a poor prognosis.

It was reported that chronic kidney disease (CKD) could increase postoperative complications and worsen the OS for patients who accepted radical surgery [18–20]. CKD is usually identified and classified by the glomerular filtration rate (GFR) [21]. Besides GFR, when the glomerular filtration function began to deteriorate, blood urea nitrogen (BUN) [22], cystatin C (CysC) [23], and serum uric acid (UA) [24] were also elevated. What's more, the changes in CysC and serum UA were more sensitive and prominent than serum creatinine in the early period when glomerular filtration function was impaired [25]. As a result, we deduced that BUN, UA, and CysC might be related to the short-term outcomes and prognosis for CRC patients undergoing radical resection as well.

Both CysC and UA were proved to be interacted with tumor development and invasion. Previous studies reported the CySc was a marker for the prognosis of urinary system carcinoma [26, 27], esophageal cancer [28], and lung cancer patients [29]. Only Kos J et al. reported that CRC patients, after surgery with high cystatin C, had lower survival [30]. Similarly, the level of UA in the serum was correlated with the survival of patients with pancreatic cancer [31], laryngeal cancer [32], and so on, but its specific role in the prognosis for CRC patients remained controversial. Meanwhile, little was known about the predictive value of these factors for short-term outcomes.

As a result, the current study was designed to investigate the impact of BUN, CysC, and UA in serum on the short-term outcomes and prognosis of CRC patients undergoing radical surgery.

## 2. Materials and Methods

### 2.1. Patients

Patients who underwent radical CRC surgery were included from Jan 2011 to Jan 2020 in a single clinical center. The study was approved by the ethics committee of our institution (the First Affiliated Hospital of Chongqing Medical University, 2022-135-2), and all patients signed informed consent forms. This study was conducted in accordance with the World Medical Association Declaration of Helsinki as well.

### 2.2. Inclusion and Exclusion Criteria

**Figure 1:**
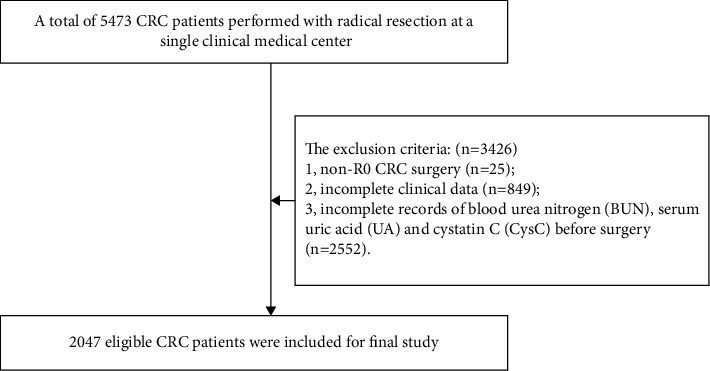
Flowchart for patient selection.

Patients who underwent radical CRC surgery were included (*n* = 5473). The exclusion criteria were as follows: 1, non-R0 surgery (*n* = 25); 2, incomplete clinical data (*n* = 849); and 3, incomplete records of BUN, UA, and CysC before surgery (*n* = 2552). Finally, a total of 2047 CRC patients were included in this study (Figure 1).

### 2.3. Data Collection

The values of BUN, UA, and CysC were determined by the blood tests conducted a week before surgery. The baseline characteristics collected were as follows: age, sex, body mass index (BMI), smoking, drinking, hypertension, type 2 diabetes mellitus (T2DM), coronary heart disease (CHD), surgical method, tumor location, tumor node metastasis (TNM) stage, and tumor size. The short-term outcomes included operation time, intraoperative blood loss, postoperative hospital stay, overall complications, and major complications. The long-term prognosis was estimated by the OS and DFS. All the data were collected from the electronic medical record system, outpatient visits, and telephone interviews.

### 2.4. Definitions

The TNM stage was identified according to the AJCC 8th Edition [33]. The postoperative complications were classified on the basis of the Clavien-Dindo classification [34], and major complications were regarded as ≥ grade III. OS was defined as the time from surgery to death or loss of follow-up. DFS was calculated from the date of surgery to the date of recurrence or death.

### 2.5. Treatment and Follow-Up

All patients underwent radical surgery according to standard principles, and R0 resection was confirmed by pathology. Patients were regularly followed up every six months in the first three years and every year in the next years.

### 2.6. Optimal Cut-Off and Groups

According to the upper limits of the reference ranges of BUN, UA, and CysC, patients were divided into the abnormal BUN group (BUN>8.2 mmol/L) and the normal BUN group (BUN≤8.2 mmol/L); the abnormal UA group (UA>357 *μ*mol/L) and the normal UA group (UA≤357 *μ*mol/L); as well as the abnormal CysC group (CysC>1.09 mg/L) and the normal CysC group (CysC≤1.09 mg/L).

### 2.7. Statistical Analysis

A normality test was performed on the measurement data. The measurement data conforming to the normal distribution were expressed as mean ± standard deviation (SD), and an independent-sample*t*-test was used to compare the indicators between groups; the measurement data not conforming to the normal distribution were expressed as the median (minimum value and maximum value), and a Mann−Whitney *U* test was adopted for comparison between groups. Categorical variables are expressed as absolute values and percentages, and Chi-square test or Fisher's exact test was performed. The Kaplan−Meier method was used to estimate the OS and DFS, and a log-rank test was conducted to compare the OS and DFS between the CysC groups in different tumor stages. Moreover, Cox regression analysis was performed to identify independent risk factors for OS and DFS. Data were analyzed using SPSS (version 22.0) statistical software. A bilateral *p* value of <0.05 was considered statistically significant.

## 3. Results

### 3.1. Patients and Characteristics

A total of 2047 CRC patients who underwent radical resection were included in the current study, and these patients were divided into different groups according to the values of BUN, UA, and CysC.

As a result, there were 1937 patients in the normal BUN group and 110 patients in the abnormal BUN group. The abnormal BUN group had an older age (*p* < 0.01), more males (*p* < 0.01), higher portion of smoking (*p*=0.001), drinking (*p*=0.004), hypertension (*p* < 0.01), and T2DM (*p*=0.001) than the normal BUN group (Table 1).

Similarly, 1756 patients were in the normal UA group, and 291 patients were in the abnormal UA group. The abnormal UA group had an older age (*p*=0.009), a higher BMI (*p* < 0.01), higher incidence of hypertension (*p* < 0.01) and CHD (*p*=0.038), and more tumor size< 5 cm (*p*=0.016). (Table 2).

Moreover, 1627 patients and 420 patients were included in the normal CysC group and the abnormal CysC group, respectively. The abnormal CysC group had older age (*p* < 0.01), more males (*p* < 0.01), a higher portion of smoking (*p* < 0.01), and drinking (*p*=0.013), a higher incidence of hypertension (*p* < 0.01), T2DM (*p*=0.017), and CHD (*p* < 0.01), more open surgery (*p* < 0.01). (Table 3).

### 3.2. Short-Term Outcomes

The short-term outcomes were compared in different groups. Accordingly, no difference was found between the normal UA group and the abnormal UA group (*p* > 0.05). Patients in the abnormal BUN group had a longer hospital stay (*p*=0.002) and more overall complications (*p*=0.001) than the normal BUN group. The abnormal CysC group had a longer hospital stay (*p* < 0.01), more overall complications (*p* < 0.01), and more major complications (*p*=0.001) than the normal CysC group (Tables 1–3).

### 3.3. Cox Analyses for OS and DFS

Cox regression analyses were conducted to identify the independent risk factors for OS and DFS. As a consequence, age (*p* < 0.01, HR = 1.039, 95% CI = 1.028–1.050), sex (*p*=0.009, HR = 0.716, 95% CI = 0.558–0.919), tumor stage (*p* < 0.01, HR = 2.123, 95% CI = 1.823–2.473), smoking (*p*=0.012, HR = 1.356, 95% CI = 1.070–1.717), tumor size (*p*=0.002, HR = 1.451, 95% CI = 1.147–1.837), CysC (*p*=0.006, HR = 1.441, 95% CI = 1.108–1.875), and overall complications (*p* < 0.01, HR = 1.682, 95% CI = 1.311–2.158) were potential risk factors for OS. In multivariate analysis, age (*p* < 0.01, HR = 1.041, 95% CI = 1.029–1.053), tumor stage (*p* < 0.01, HR = 2.134, 95% CI = 1.828–2.491), and overall complications (*p*=0.002, HR = 1.499, 95% CI = 1.166–1.928) were independent risk factors for OS (Table 4).

As for DFS, age (*p* < 0.01, HR = 1.026, 95% CI = 1.017–1.036), sex (*p*=0.044, HR = 0.797, 95% CI = 0.639–0.994), tumor stage (*p* < 0.01, HR = 2.053, 95% CI = 1.791–2.352), smoking (*p*=0.020, HR = 1.288, 95% CI = 1.041–1.594), tumor size (*p* = 0.007, HR = 1.340, 95% CI = 1.084–1.656), CysC (*p*=0.012, HR = 1.357, 95% CI = 1.068–1.723), and overall complications (*p* < 0.01, HR = 1.542, 95% CI = 1.227–1.937) were potential indicators. Furthermore, age (*p* < 0.01, HR = 1.026, 95% CI = 1.016–1.037), tumor stage (*p* < 0.01, HR = 2.053, 95% CI = 1.788–2.357), and overall complications (*p*=0.002, HR = 1.440, 95% CI = 1.144–1.814) were independent risk factors (Table 5).

However, none of BUN, CysC, or UA were independent risk factors for OS or DFS (*p* > 0.05).

### 3.4. Kaplan−Meier Curves in Different TNM Stages

**Figure 2:**
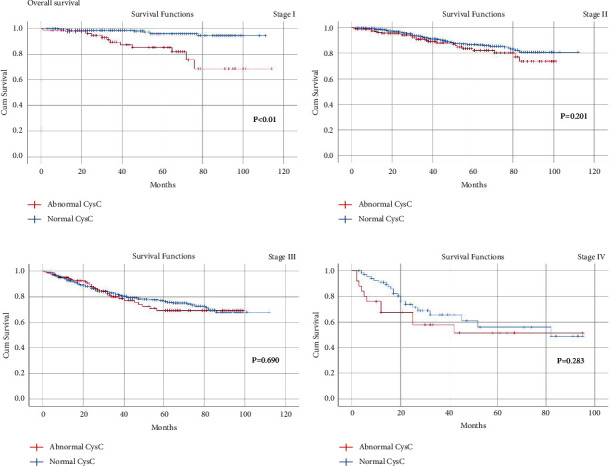
Kaplan−Meier survival curve for the impact of preoperative CysC on the overall survival of patients in TNM stages I-IV.

**Figure 3:**
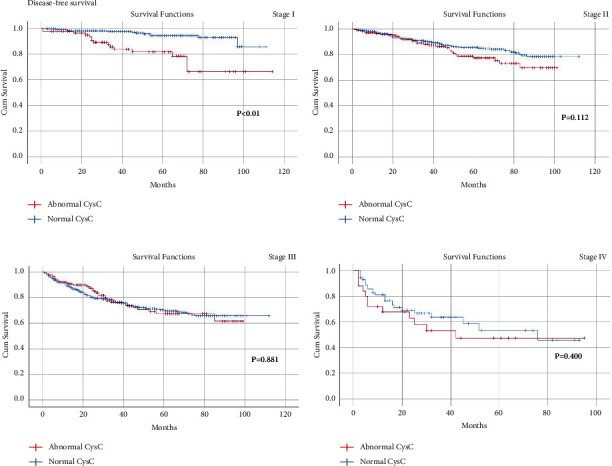
Kaplan−Meier survival curve for the impact of preoperative CysC on the disease-free survival of patients in TNM stages I-IV.

The median follow-up time was 35 (1–114) months. Since CysC was found to be a potential risk factor for OS and DFS, we adopted the Kaplan−Meier method and log-rank test to compare the OS (Figure 2) and DFS (Figure 3) between the abnormal CysC group and the normal CysC group in TNM stages I–IV. Consequently, abnormal CysC were associated with worse OS (*p* < 0.01) and DFS (*p* < 0.01) for CRC patients in TNM stage I. However, no significant difference was found between the two groups for OS and DFS in stages II–IV (*p* > 0.05).

## 4. Discussion

A total of 2047 CRC patients were enrolled in the current study. We investigated the impact of biochemical indicators, including BUN, UA, and CysC, which were associated with kidney function, on the short-term outcomes and prognosis of CRC patients who underwent radical surgery.

It was reported that nearly 15% of CRC patients had CKD [35]. Previous studies found that CRC patients with CKD had more postoperative complications, especially cardiovascular diseases [18–20]. The abnormal renal function also led to an increase in BUN, UA, and CysC in serum. In this study, patients in the abnormal BUN group had longer hospital stay and more overall complications than the normal BUN group, and patients in the abnormal CysC group had a longer hospital stay and more overall complications and major complications than the normal CysC group. However, we found the abnormal level of UA did not affect the short-term outcomes. The CysC was a sensitive indicator which could early identify the injury of kidney filtration function [23]. Thus, the monitoring of preoperative CysC might help to early identify patients with postoperative complication risks.

BUN was one of the main products in protein metabolism, and it was usually used to estimate glomerular filtration function [22]. The BUN in the serum began to increase only if the GFR decreased to less than 50%, which reflected the severity of CKD. Sohal DP et al. found elevated BUN before surgery indicated worse OS in pancreatic adenocarcinoma, which was simply explained as that higher BUN might imply subclinical organ dysfunction. However, whether preoperative BUN affected the prognosis of CRC patients was rarely reported, and our study found that BUN was not associated with the OS or DFS. The underlying mechanism needs to be further studied.

UA was an antioxidant as well as a pro-oxidant, which was produced from purine nucleotides, and the process was mediated by xanthine oxidase [36, 37]. It was widely reported that oxidative stress could facilitate the development of tumors; therefore, the prognostic value of UA might be controversial. Dziaman et al. first reported that CRC patients with high levels of UA in their serum had longer survival in a cohort study conducted in Poland [38]. However, in China, Mao et al. obtained the opposite conclusion that lower UA-level patients lived longer than those with higher serum UA [39]. The author attributed the incongruity to racial differences. Moreover, in a retrospective study including 332 patients, it was found that a higher preoperative UA was a risk factor for OS [40]. Nevertheless, different from the conclusions above, we found that preoperative UA had no obvious impact on OS or DFS for CRC patients.

In this study, although higher CysC was found to be associated with worse OS and DFS in CRC patients in tumor stage I, CysC was not an independent risk factor for DFS and OS. Kos demonstrated that patients with higher CysC had worse OS but it was not an independent indicator as well [30]. Besides the capacity to indicate the injury of kidney function, CysC was an inhibitor of cysteine proteinases, and the imbalance between cysteine proteinases and its inhibitors was proved to promote tumor invasion and metastasis [41]. As a result, the level of CysC in the serum might reflect the activity of tumor cells and the intensity of antitumor reactions in the body of cancer patients, which partly helped to explain the correlation between CysC and prognosis. However, it remained unclear why only patients in TNM stage I had worse OS and DFS.

To our knowledge, this was the first study to find that abnormal CysC was associated with more postoperative complications and worse OS and DFS in CRC patients with a relatively large sample size. Meanwhile, we also pointed out that preoperative UA had no obvious impact on OS and DFS for CRC patients, which was inconsistent with previous studies. Nevertheless, there were some limitations in our study as well. For this was a retrospective study conducted in a single clinical center, confounding bias was inevitable. Second, chemotherapeutic information was lacking in TNM III-IV patients, which might impair the reliability of the survival analysis. Therefore, multicenter prospective studies with a large sample size are needed to identify the predictive roles of these indicators.

In conclusion, abnormal CysC was significantly associated with worse OS and DFS at TNM stage I, and abnormal CysC and BUN were related to more postoperative complications. However, preoperative BUN and UA in the serum might not affect OS and DFS for CRC patients who underwent radical resection.

## Figures and Tables

**Table 1:** Comparison between the normal BUN group and the abnormal BUN group.

Characteristics	Normal BUN (1937)	Abnormal BUN (110)	*p* value
Age, year	63.0 (20.0–94.0)	70.0 (38.0–91.0)	<0.01^*∗*^
*Sex*			<0.01^*∗*^
Male	1124 (58.0%)	88 (80.0%)	
Female	813 (42.0%)	22 (20.0%)	
BMI, kg/m^2^	22.8 (14.2–37.3)	22.0 (15.8–33.7)	0.054
Smoking	726 (37.5%)	59 (5act3.6%)	0.001^*∗*^
Drinking	590 (30.5%)	48 (43.6%)	0.004^*∗*^
Hypertension	479 (24.7%)	50 (45.5%)	<0.01^*∗*^
T2DM	231 (11.9%)	25 (22.7%)	0.001^*∗*^
CHD	69 (3.6%)	7 (6.4%)	0.124
Open surgery	189 (9.8%)	13 (11.8%)	0.481
*Tumor location*			0.406
Colon	853 (44.0%)	44 (40.0%)	
Rectum	1084 (56.0%)	66 (60.0%)	
*TNM stage*			0.582
I	391 (20.2%)	20 (18.2%)	
II	795 (41.0%)	40 (36.4%)	
III	660 (34.1%)	44 (40.0%)	
IV	91 (4.7%)	6 (5.5%)	
*Tumor size*			0.881
<5 cm	1141 (58.9%)	64 (58.2%)	
≥5 cm	796 (41.1%)	46 (41.8%)	
Operation time (min)	215.0 (45.0–695.0)	221.5 (75.0–540.0)	0.530
Blood loss (mL)	50.0 (5.0–3500.0)	80.0 (5.0–2200.0)	0.335
Hospital stay (day)	9.0 (2.0–269.0)	10.0 (4.0–54.0)	0.002^*∗*^
Overall complications	399 (20.6%)	37 (33.6%)	0.001^*∗*^
Major complications	46 (2.4%)	3 (2.7%)	0.745

Variables are expressed as the median and range, *n* (%), ^*∗*^*p*-value <0.05. Abbreviations: BUN, blood urea nitrogen; T2DM, type 2 diabetes mellitus; BMI, body mass index; CHD, coronary heart disease.

**Table 2:** Comparison between the normal UA group and the abnormal UA group.

Characteristics	Normal UA (1756)	Abnormal UA (291)	*p* value
Age, year	63.0 (20.0–93.0)	65.0 (30.0–94.0)	0.009^*∗*^
*Sex*			0.185
Male	1050 (58.9%)	162 (55.7%)	
Female	706 (40.2%)	129 (44.3%)	
BMI, kg/m^2^	22.5 (14.2–36.7)	23.9 (14.7–37.3)	<0.01^*∗*^
Smoking	677 (38.6%)	108 (37.1%)	0.640
Drinking	549 (31.3%)	89 (30.6%)	0.817
Hypertension	422 (24.0%)	107 (36.8%)	<0.01^*∗*^
T2DM	211 (12.0%)	45 (15.5%)	0.100
CHD	59 (3.4%)	17 (5.8%)	0.038^*∗*^
Open surgery	173 (9.9%)	29 (10.0%)	0.952
*Tumor location*			0.654
Colon	773 (44.0%)	124 (42.6%)	
Rectum	983 (56.0%)	167 (57.4%)	
*TNM stage*			0.542
I	347 (19.8%)	64 (22.0%)	
II	721 (41.1%)	114 (39.2%)	
III	601 (34.2%)	103 (35.4%)	
IV	87 (5.0%)	10 (3.4%)	
*Tumor size*			0.016^*∗*^
<5 cm	1015 (57.8%)	190 (65.3%)	
≥5 cm	741 (42.2%)	101 (34.7%)	
Operation time (min)	215.0 (45.0–695.0)	217.0 (70.0–560.0)	0.752
Blood loss (mL)	50.0 (5.0–3500.0)	50.0 (5.0–1500.0)	0.441
Hospital stay (day)	9.0 (2.0–97.0)	9.0 (3.0–269.0)	0.950
Overall complications	365 (20.7%)	71 (24.4%)	0.163
Major complications	40 (2.3%)	9 (3.1%)	0.400

Variables are expressed as the median and range, *n* (%), ^*∗*^*p*-value <0.05. Abbreviations: UA, uric acid; T2DM, type 2 diabetes mellitus; BMI, body mass index; CHD, and coronary heart disease.

**Table 3:** Comparison between the normal CysC group and the abnormal CysC group.

Characteristics	Normal CysC (1627)	Abnormal CysC (420)	*p* value
Age, year	61.0 (20.0–91.0)	71.5 (37.0–94.0)	<0.01^*∗*^
*Sex*			<0.01^*∗*^
Male	909 (55.8%)	303 (72.1%)	
Female	718 (44.1%)	117 (27.9%)	
BMI, kg/m^2^	22.8 (14.7–37.3)	22.6 (14.2–35.4)	0.762
Smoking	585 (35.9%)	200 (47.6%)	<0.01^*∗*^
Drinking	486 (29.9%)	152 (36.2%)	0.013^*∗*^
Hypertension	354 (21.8%)	175 (41.7%)	<0.01^*∗*^
T2DM	189 (11.6%)	67 (16.0%)	0.017^*∗*^
CHD	48 (3.0%)	28 (6.7%)	<0.01^*∗*^
Open surgery	141 (8.7%)	61 (14.5%)	<0.01^*∗*^
*Tumor location*			0.655
Colon	717 (44.1%)	180 (42.9%)	
Rectum	910 (55.9%)	240 (57.1%)	
*TNM stage*			0.613
I	329 (20.2%)	82 (19.5%)	
II	667 (41.0%)	168 (40%)	
III	559 (34.3%)	145 (34.5%)	
IV	72 (4.4%)	25 (6.0%)	
*Tumor size*			0.141
<5 cm	971 (59.7%)	234 (55.7%)	
≥5 cm	656 (40.3%)	186 (44.3%)	
Operation time (min)	215.0 (45.0–695.0)	220.0 (86.0–560.0)	0.454
Blood loss (mL)	50.0 (5.0–3500.0)	70.0 (5.0–2200.0)	0.088
Hospital stay (day)	9.0 (3.0–70.0)	10.0 (2.0–269.0)	<0.01^*∗*^
Overall complications	307 (18.9%)	129 (30.7%)	<0.01^*∗*^
Major complications	30 (1.8%)	19 (4.5%)	0.001^*∗*^

Variables are expressed as the median and range, *n* (%), ^*∗*^*p*-value <0.05. Abbreviations: CysC, cystatin C; T2DM, type 2 diabetes mellitus; BMI, body mass index; CHD, and coronary heart disease.

**Table 4:** Univariate and multivariate analyses of overall survival.

Risk factors	*Univariate analysis*		*Multivariate analysis*	
	HR (95% CI)	*p* value	HR (95% CI)	*p* value
Age (years)	1.039 (1.028–1.050)	<0.01^*∗*^	1.041 (1.029–1.053)	<0.01^*∗*^
Sex (female/male)	0.716 (0.558–0.919)	0.009^*∗*^	0.818 (0.598–1.119)	0.208
BMI (kg/m^2^)	0.969 (0.933–1.007)	0.105		
T2DM (yes/no)	1.303 (0.925–1.836)	0.130		
Tumor site (colon/rectum)	1.011 (0.796–1.283)	0.931		
Tumor stage (IV/III/II/I)	2.123 (1.823–2.473)	<0.01^*∗*^	2.134 (1.828–2.491)	<0.01^*∗*^
Smoking (yes/no)	1.356 (1.070–1.717)	0.012^*∗*^	1.211 (0.899–1.630)	0.207
Drinking (yes/no)	1.190 (0.929–1.525)	0.169		
Hypertension (yes/no)	0.975 (0.739–1.285)	0.855		
CHD (yes/no)	1.590 (0.928–2.722)	0.091		
Tumor size (≥5 cm/<5 cm)	1.451 (1.147–1.837)	0.002^*∗*^	1.231 (0.972–1.560)	0.085
BUN (abnormal/normal)	1.348 (0.855–2.125)	0.198		
UA (abnormal/normal)	0.712 (0.485–1.046)	0.083		
CysC (abnormal/normal)	1.441 (1.108–1.875)	0.006^*∗*^	0.860 (0.645–1.147)	0.304
Overall complications (yes/no)	1.682 (1.311–2.158)	<0.01^*∗*^	1.499 (1.166–1.928)	0.002^*∗*^

^*∗*^ *p*-value <0.05. Abbreviations: BUN, blood urea nitrogen; UA, uric acid; CysC, cystatin C; HR, hazard ratio; CI, confidence interval; BMI, body mass index; T2DM, and type 2 diabetes mellitus.

**Table 5:** Univariate and multivariate analyses of disease-free survival.

Risk factors	*Univariate analysis*		*Multivariate analysis*	
	HR (95% CI)	*p* value	HR (95% CI)	*p* value
Age (years)	1.026 (1.017–1.036)	<0.01^*∗*^	1.026 (1.016–1.037)	<0.01^*∗*^
Sex (female/male)	0.797 (0.639–0.994)	0.044^*∗*^	0.904 (0.682–1.199)	0.484
BMI (kg/m^2^)	0.992 (0.959–1.026)	0.634		
T2DM (yes/no)	1.157 (0.843–1.589)	0.366		
Tumor site (colon/rectum)	1.024 (0.827–1.268)	0.829		
Tumor stage (IV/III/II/I)	2.053 (1.791–2.352)	<0.01^*∗*^	2.053 (1.788–2.357)	<0.01^*∗*^
Smoking (yes/no)	1.288 (1.041–1.594)	0.020^*∗*^	1.189 (0.906–1.561)	0.212
Drinking (yes/no)	1.206 (0.965–1.507)	0.100		
Hypertension (yes/no)	1.021 (0.800–1.303)	0.868		
CHD (yes/no)	1.421 (0.860–2.348)	0.170		
Tumor size (≥5 cm/<5 cm)	1.340 (1.084–1.656)	0.007^*∗*^	1.134 (0.917–1.404)	0.247
BUN (abnormal/normal)	1.115 (0.717–1.734)	0.629		
UA (abnormal/normal)	0.839 (0.608–1.159)	0.287		
CysC (abnormal/normal)	1.357 (1.068–1.723)	0.012^*∗*^	0.941 (0.725–1.223)	0.651
Overall complications (yes/no)	1.542 (1.227–1.937)	<0.01^*∗*^	1.440 (1.144–1.814)	0.002^*∗*^

^*∗*^ *p* value <0.05. Abbreviations: BUN, blood urea nitrogen; UA, uric acid; CysC, cystatin C; HR, hazard ratio; CI, confidence interval; BMI, body mass index; T2DM, and type 2 diabetes mellitus.

## Data Availability

The data used to support the findings of this study are available from the corresponding author upon request.
